# USP14 inhibition enhances Parkin-independent mitophagy in iNeurons

**DOI:** 10.1016/j.phrs.2024.107484

**Published:** 2024-10-30

**Authors:** Greta Bernardo, Miguel A. Prado, Anna Roshani Dashtmian, Mariavittoria Favaro, Sofia Mauri, Alice Borsetto, Elena Marchesan, Joao A. Paulo, Steve P. Gygi, Daniel J. Finley, Elena Ziviani

**Affiliations:** a Department of Biology, University of Padova, Padova, Italy; b Department of Cell Biology, Harvard Medical School, Boston, USA; c Instituto de Investigación Sanitaria del Principado de Asturias (ISPA), Oviedo, Spain

**Keywords:** USP14, UPS, Autophagy, Mitophagy, PINK1, Parkin, MARCH5/MITOL

## Abstract

Loss of proteostasis is well documented during physiological aging and depends on the progressive decline in the activity of two major degradative mechanisms: the ubiquitin-proteasome system (UPS) and the autophagy-lysosomal pathway. This decline in proteostasis is exacerbated in age-associated neurodegenerative diseases, such as Parkinson’s Disease (PD). In PD, patients develop an accumulation of aggregated proteins and dysfunctional mitochondria, which leads to ROS production, neuroinflammation and neurodegeneration. We recently reported that inhibition of the deubiquitinating enzyme USP14, which is known to enhance both the UPS and autophagy, increases lifespan and rescues the pathological phenotype of two *Drosophila* models of PD. Studies on the effects of USP14 inhibition in mammalian neurons have not yet been conducted. To close this gap, we exploited iNeurons differentiated from human embryonic stem cells (hESCs), and investigated the effect of inhibiting USP14 in these cultured neurons. Quantitative global proteomics analysis performed following genetic ablation or pharmacological inhibition of USP14 demonstrated that USP14 loss of function specifically promotes mitochondrial autophagy in iNeurons. Biochemical and imaging data also showed that USP14 inhibition enhances mitophagy. The mitophagic effect of USP14 inhibition proved to be PINK1/Parkin- independent, instead relying on expression of the mitochondrial E3 Ubiquitin Ligase MITOL/MARCH5. Notably, USP14 inhibition normalized the mitochondrial defects of Parkin KO human neurons.

## 1. Introduction

In the last two decades, increasing life expectancy and decline of fertility are fueling an exponential increase of the population over 65 years of age[1]. As a result, the occurrence of age-related neurodegenerative diseases such as Parkinson’s Disease (PD), Alzheimer’s Disease (AD) and Amyotrophic Lateral Sclerosis (ALS) is expected to increase. Despite great efforts from the scientific community worldwide, there is no cure for these devastating diseases, and to date only a few pharmaceutical treatments, able to moderate the symptoms or delay the neurodegeneration process, have been developed[2].

The pathogenesis and molecular basis of most neurodegenerative disorders remains largely unclear. However, multiple underlying mechanisms have been proposed; some are specific for each disease and lead to the degeneration of specific subclasses of neurons; others are shared between the different disorders, and include mitochondrial dysfunction, neuroinflammation, oxidative stress, and protein aggregation[3–5]. Mitochondria dysfunction in particular has been linked to neurodegeneration and neurodegenerative diseases since early studies [6]. In neurodegenerative diseases, every aspect of mitochondrial physiology seems to be disrupted[7–9], with several case studies reporting mitochondrial dysfunction[10–13], and specific impairment of mitochondrial Complex I [14–18].

Further evidences of a key link between PD in particular and mitochondrial damage come from genetic studies. Two genes involved in the degradation of dysfunctional mitochondria via autophagy, PINK1 and Parkin, are mutated in juvenile forms of autosomal recessive Parkinsonism[19–21], providing a direct association between aberrant mitophagy and PD onset. Follow up studies strengthened the prominent role of mitochondrial quality control in neurodegenerative processes [22], with several study cases gathering substantial evidences that mitophagy serves as a shared mechanism, which impairment is central to the pathology of PD[23–25].

Mitophagy initiation involves crosstalk between the two major degradative systems, the Ubiquitin Proteasome System (UPS) and the autophagy-lysosomal pathway, which collectively maintain cellular homeostasis through the detection and degradation of misfolded proteins, aggregated proteins, and dysfunctional organelles[26]. The fundamental signaling molecule that links these two pathways is ubiquitin, a small 8.5-kDa protein that is covalently attached to Lys residues of target proteins to modulate their fate[27]. In a simplified view, ubiquitinated proteins on the mitochondrial surface are recognized by the UPS, which mediate their degradation, and autophagic receptors, which promote autophagosome assembly and delivery to the lysosome [28].

Protein ubiquitination is a dynamic and reversible process controlled by two types of enzymes: ubiquitin ligases and deubiquitinating enzymes (DUBs). Several ubiquitin E3 ligases have been associated with the regulation of mitophagy but the best characterized is the E3 ubiquitin ligase Parkin. Parkin, in conjunction with the protein kinase PINK1, controls a surveillance pathway for the detection and removal of dysfunctional mitochondria through ubiquitin-dependent mitophagy [29,30]. In a nutshell, PINK1 is imported into functional mitochondria, cleaved in the inner mitochondrial membrane, translocated back to the cytoplasmic space, and rapidly degraded[31,32]. Following a mitophagy stimulus, PINK1 is stabilized on the outer mitochondrial membrane (OMM) where it is activated through autophosphorylation; once activated, PINK1 induces Parkin translocation to the OMM, and its ubiquitin E3-ligase activity by phosphorylating Parkin as well as pre-existing ubiquitin chains on OMM proteins[33–35]. Parkin ubiquitinates OMM proteins creating new substrates for PINK1 phosphorylation, thereby leading to a feed-forward mechanism, which amplifies the mitophagic signal[33,36–39]. Ubiquitin chains formed by Parkin on the OMM display linkage types typical of both autophagy and proteasome-dependent degradation[38,40].

The physiological relevance of the PINK1/Parkin-dependent mitophagy pathway in neuronal cells is controversial due to the minimal phenotype in mice deriving from germline deletion of PINK1[41] or Parkin[42]. Such mice fail to show PD-like phenotypes, such as loss of dopaminergic (DA) neurons and motor defects[41,42], and do not develop any impairment in basal mitophagy[43], suggesting that alternative pathways during development likely compensate for PINK1 and Parkin loss. PINK1/Parkin depletion generates PD-like phenotypes, including loss of DA neurons and motor defects, in *Drosophila* models [44,45], where genetic redundancy is less prominent, and compensatory mechanisms during development are presumably lacking. Similar phenotype has been observed in conditional KO models in which Parkin is deleted in adult mice[46]. These findings support the hypothesis that compensatory mechanisms are likely to moderate the physiological impact of PINK1/Parkin loss during development, and that PINK1/Parkin-dependent mitophagy might become relevant to counteract neurodegeneration in the presence of pathological stimuli specifically affecting mitochondrial physiology[47,48]. However, while some studies showed that Parkin KO mice subjected to mitochondrial stress (exhaustive exercise, increased mtDNA damage)[49,50] develop an obvious Parkinsonian phenotype, another recent work failed to reproduce these results, and did not see any synergistic effect between Parkin loss and mitochondrial dysfunction[51]. At present it is not clear whether mitochondrial dysfunction is a consequence of neurodegeneration or actively contributes to neuronal cell death. Nevertheless, these studies collectively highlight mitochondrial dysfunction as prominent hallmark, and the existence of alternative mitophagic pathways that compensate for PINK1 and Parkin loss.

In this scenario, stimulation of these alternative mitophagic pathways represents an attractive approach to promote basal mitochondrial homeostasis through accelerated selective clearance of damaged or aged mitochondria.

One way to activate basal mitophagy is by acting on DUBs; these enzymes oppose E3 ubiquitin ligases by eliminating ubiquitin chains from targeted proteins or entire organelles, such as mitochondria, thus preventing their elimination through the UPS and autophagy-lysosome pathway[52,53]. Among these DUBs, the proteasome-associated USP14 is a particularly appealing target for its capacity to modulate both the UPS[54,55] and autophagy[56–58], and for the cytoprotective effect of its inhibition highlighted in several studies [59–65]. Chemical inhibition of USP14 triggers an enhancement of proteasome activity, and degradation of different substrates in cell culture models [59,62,66]. USP14 also negatively regulates autophagy by removing K63 ubiquitin chains from Beclin1, a regulatory element in the Beclin1/VPS34 complex necessary for autophagosome nucleation[56]. Accumulation of aggregated insoluble proteins and dysfunctional organelle are key hallmarks in neurodegenerative conditions[67]. Inhibition of USP14, which correlates with proteasome activation[54,55] and autophagy enhancement[56,68], increases the proteolytic capacity of the cell, and it is expected to be beneficial in neurodegenerative conditions characterized by intracytoplasmic deposition of insoluble aggregates. Indeed, inhibition of USP14 with specific inhibitor IU1–47 enhances Tau degradation in cultured neurons, with no reported toxicity for neurons [59]. Another study found that inhibition of USP14 with inhibitor IU1 is protective against neuronal cell death caused by ischemic stroke associated with over-production of misfolded and aggregating proteins[61]. We also recently reported that pharmacological and genetic inhibition of USP14 extended the lifespan of two *in vivo Drosophila* models of PD, the PINK1 and Parkin KO flies[68]. Inhibition of USP14 rescued climbing behavior of these flies[68], as well as non-motor phenotype[69], and restored mitochondria function and ultrastructure[68]. The protective effect of USP14 inhibition can be ascribed at least in part to its mitophagic effect, which we demonstrated in different cell lines[68].

As highlighted in the abovementioned studies, a series of potent and highly selective inhibitors are available for USP14, making it an ideal candidate for potential therapeutic development. The first described inhibitor, IU1 (IC_50_ = 4–5 μM), was developed in the Finley and King laboratories in 2010[55]. The same group synthesized a new compound called IU1–47, a derivative of IU1, which is tenfold more potent (IC_50_ =0.6 μM), and completely benign for neuronal cells [59].

At present, studies on the effects of USP14 inhibition in neurons of human origin have not yet been conducted. Thus, we took advantage of a recently generated line of human embryonic stem cells (hESCs) able to rapidly and efficiently differentiate into functional iNeurons[70,71], and tested the effect of USP14 inhibition. We found that USP14 inhibition promoted PINK1/Parkin-independent mitophagy, and that the mitophagic effect depended on the expression of the mitochondrial E3 ubiquitin ligase MARCH5/MITOL. Importantly, USP14 inhibition completely recovered the mitochondrial phenotype associated with Parkin KO iNeurons.

## 2. Materials and methods

All details and catalogue numbers can be found in the Materials Table.

### 2.1. Cell culture and iNeurons differentiation

Human SH-SY5Y neuroblastoma, mouse embryonic fibroblast (MEFs) cells and Human Embryonic Kidney cells (HEK293) were maintained as adherent in Dulbecco’s Modified Eagle Medium (DMEM) culture media supplemented with the 10 % (v/v) heat-inactivated fetal bovine serum (FBS), 2 mM L-glutamine, 1X MEM Non-Essential Amino Acids Solution (100X) and 1X Penicillin-Streptomycin solution (100X). The medium and all supplements were purchased from Thermo Fisher Scientific. H9 hESCs were cultured in TeSR^™^-E8^™^ medium on Matrigel-coated tissue culture plates with daily medium change. All cells were cultured at 37 °C and in a 5 % CO2 humidified atmosphere. H9 cells were passaged every 4–5 days with 0.5 mM EDTA in DMEM/F12. Introduction of the TRE3G-NGN2 insert into the AAVS1 site, necessary for iNeurons differentiation, and gene editing to obtain the desired mutants (PINK1 KO, Parkin KO, USP14 KO, MUL1 KO, MARCH5 KO, and BNIP3L KO) was performed by the gene editing core facility in the Dept. of Cell Biology at Harvard Medical School. The cells were kindly provided by Prof. Wade Harper (HMS, Dept. of Cell Biology). For details on the gene editing methods see refs[70–72]. For H9 hESCs conversion to iNeurons, cells were treated with Accutase and plated on Matrigel-coated tissue plates in DMEM/F12 supplemented with 1x N2, 1x NEAA, human brain-derived neurotrophic factor (BDNF, 10 ng/ml), human Neurotrophin-3 (NT-3, 10 ng/l), human recombinant laminin (0.2 mg/ml), Y-27632 (10 mM) and Doxycycline (2 mg/ml) on Day 0. On Day 1, Y-27632 was withdrawn. On Day 2, medium was replaced with Neurobasal medium supplemented with 1x B27 and 1x Glutamax containing BDNF, NT-3 and 2 mg/ml Doxycycline. Starting on Day 4, half of the medium was replaced every other day thereafter. On Day 7, the cells were treated with Accutase and plated on Matrigel-coated tissue plates. Doxycycline was withdrawn on Day 10. We and others previously performed in deep characterization of iNeurons neuronal class and maturity, which indicate that the iNeurons are mature from day 11 upon starting the differentiation protocol [73,75]. Treatments and experiments were performed between day 11 and 13.

### 2.2. Generation of stable mitophagic flux reporters hESC lines

H9 hESC harbouring the mitochondrial matrix mCherry-GFP flux reporter were generated by transfection of 1×10^5^ cells with 1 μg pAC150-PiggyBac-matrix-mCherry-eGFPXL (Harper lab) and 1 μg pCMV-HypBAC-PiggyBac-Helper (Sanger Institute) in conjunction with the transfection reagent FuGENE HD. The cells were selected and maintained in TeSR^™^-E8^™^ medium supplemented with 200 mg/ml Hygromycin. Hygromycin was kept in the medium during differentiation to iNeurons.

### 2.3. Compounds and treatments

Cells were treated in the corresponding cell culture medium. USP14 inhibition was performed using IU1 and IU1–47 inhibitor for 24–48 hours at different final concentrations, as reported in the figure’s legend. The concentration and duration of IU1 treatments was chosen based on previous results obtained in different cell lines[68]. Sub-toxic concentrations of IU1–47 inhibitor were determined by using a cell viability assay (MTT test, please see below). Concentration and duration of the treatment was determined based on IU1–47 IC_50_ (0.6μM) as well as information found in the literature, which show that 24 hours treatments are sufficient for IU1–47 to exert its inhibitory function[58,59]. Antimycin/Oligomycin were used in combination as a positive control to induce stress-mediated mitophagy. The late-stage autophagy inhibitor Bafilomycin A was used at a final concentration of 10 nM as a control for the blockage of the autophagic flux. DMSO was used for control conditions.

### 2.4. MTT cell viability assay

To assess cytotoxicity and cell tolerance upon treatment with IU1–47, we used the 3-(4,5-dimethylthiazol-2yl)-2,5-diphenyltetrazolium bromide (MTT) tetrazolium assay. To perform this assay, iNeurons and SH-SY5Y cells were seeded on 96-well plates and then treated with different concentrations (5, 10, 20, 50, 75, 100 or 200 μM) of IU1–47 for 24–48 hours. The range of concentrations of IU1–47 that we tested was based on the molecule IC_50_ (0.6μM). As reported in previous publication[59], the inhibitor was not toxic for neuronal cells, and it elicited some degree of toxicity (20 % cell death) at 20μM and higher (please see Supplementary Figure 1A–B). After 24–48 hours, 10 μL of MTT solution (12 mM) was added to each well and the plate was incubated at 37° C for 4 hours. The formazan crystal formed were subsequently dissolved with 50 μL DMSO per well and the absorbance at 560 nm was acquired after an incubation of 10 min at 37°C using a multi-well spectrophotometer.

### 2.5. Immunoblotting

At the indicated times, hES cells or iNeurons were lysate in RIPA buffer (140 mM NaCl; 65 mM Tris-HCl [pH 7.4]; 1 % NP-40; 0.25 % NaDeoxycholate, 1 mM EDTA, 1x protease Inhibitor Cocktail, 1xPhosSTOP phosphatase inhibitor cocktail). Protein concentration was determined by BCA assay according to manufacturer’s instructions. 30 μg of proteins were resuspended in 1xLDS with 100 mM DTT and heated for 5 min at 70°C. Equal amounts of protein and volume were loaded and run on homemade 15 % Tris-glycine SDS-Polyacrylamide gels (for LC3I and II detection) or on 4 %-20 % Bis-Tris ExpressPlus^™^ PAGE Gels. Gels were transferred via semi-dry Trans Blot Turbo transfer system for 30 min at 25 V onto PVDF membrane for immunoblotting. PVDF membranes were blocked for 1 hour at RT in 5 % BSA in TTBS (0.5 M Tris-HCl pH 7.4; 1.5 M NaCl; Tween 20 0.05 %(v/v)) and subsequently incubated with the desired primary antibody diluted in 1 % BSA in TTBS overnight at 4°C. For detection, membranes were washed 3–4 times for 10 min with TTBS and then incubated 1 h at room temperature with polyclonal horseradish-peroxidase (HRP)-conjugated secondary antibodies followed by 3 TTBS washes. Immunoreactivity was detected with Luminata Forte Western HRP substrate and images were acquired using the ImageQuant LAS 4000 instrument. Images from Western Blots were exported and analysed using ImageJ/FiJi[76].

### 2.6. RealTime qPCR

Total RNA to assess expression levels of PGC1-α and TFAM was extracted from the cells using ReliaPrep RNA Cell Miniprep isolation kit (Promega). Total DNA to evaluate relative mitochondrial DNA content was extracted from cells using the Puregene Core Kit A (Quiagen) according to manufacturer instruction. HOT FIREPol SolisGreen qPCR mix (Solis BioDyne) was used for real-time PCR with the following conditions: 95°C 10 min/40 cycles (95°C 15 sec, 60°C 1 min). Dissociation curve was generated for checking the amplification specificity of all the utilized primers (see Materials Table). The data were analyzed by comparative CT method[77] to determine fold differences in expression of target genes with respect to the internal control. For determination of mtDNA copy number, 4,5 ng of DNA were used as templates for real-time quantitative PCR procedure. Relative quantities of mtDNA were calculated as described by Bryant et al., 2022[78].

### 2.7. Measurement of Oxygen Consumption Rate (SeaHorse assay)

OCR was measured using a Seahorse XFe24 (Agilent Technologies) running Wave Controller Software 2.6 according to manufacturer’s manual. 3.5 ×10^4^ iNeurons were plated in Matrigel-coated Seahorse XF24 V7 PS cell culture microplates (Agilent) in appropriate growth or differentiation medium at day 7 of differentiation. The day before the assay cells were treated with 5 μM IU1–47 for 24 hours. On the day of assay, cell culture medium was removed stepwise with DMEM Base supplemented with 31.8 mM NaCl, 1 mM sodium pyruvate, 10 mM Glucose, 2 mM L-glutamine, 5 mM HEPES and equilibrated for 30 min-1hour at 37°C. All assays and drug dilutions were performed in this media. Measurements were taken for a total for 50 minutes, in 3-min periods with mixing and incubation intervals between treatments. After measurement of baseline respiration, 1.5 μM oligomycin was added in a single injection, mixed, and followed by 3 measurements. This step was repeated after the injection of 1.5 μM CCCP and 1 μM Antimycin A + 1 μM Rotenone. Protein concentration per well was determined using a BCA kit after lysis in RIPA buffer and used as normalization for OCR measurements.

### 2.8. Proteomics

#### 2.8.1. Proteomics – general sample preparation

Sample preparation of proteomic analysis of whole-cell extract from iNeurons was performed according to previously published studies[72,79,80]. Flash frozen cell pellets were lysed in 8 M urea buffer (8 M urea, 150 mM NaCl, 50 mM HEPES-NaOH [pH 7.5], 1x protease Inhibitor Cocktail, 1xPhosSTOP phosphatase inhibitor cocktail). Lysates were clarified by centrifugation at 17,000 × g for 15 min at 4°C. Protein concentration of the supernatant was quantified by BCA assay according to manufacturer’s instructions. To reduce and alkylate cysteines, 150 μg of protein was sequentially incubated with 5 mM TCEP for 30 min, 14 mM iodoacetamide for 30 min, and 10 mM DTT for 15 min. All reactions were performed at RT. Next, proteins were chloroform-methanol precipitated and the pellet resuspended in 200 mM EPPS pH 8.5. Then, the protease LysC was added at a ratio of 1:100 (LysC:protein) and the solution incubated overnight at RT. The day after, samples were further digested for 5 hours at 37°C with trypsin at 1:75 (trypsin:protein) ratio. Both digestions were performed in an orbital shaker at 1500 rpm. After digestion, samples were clarified by centrifugation at 17,000 × *g* for 10 min. Peptide concentration of the supernatant was quantified using a quantitative colorimetric peptide assay.

#### 2.8.2. Proteomics – quantitative proteomics using TMT

Tandem mass tag labeling of each sample was performed using the TMT kit (Thermo Fisher Scientific) [70–72,79]. Briefly, 25 μg of peptides was brought to 1 μg/μl with 200 mM EPPS (pH 8.5), acetonitrile (ACN) was added to a final concentration of 30 % followed by the addition of 50 μg of each TMT reagent. After 1 h of incubation at RT, the reaction was stopped by the addition of 0.3 % hydroxylamine for 15 min at RT. After labelling, samples were combined, desalted with tC18 SepPak solid-phase extraction cartridges (Waters), and dried in the SpeedVac. Next, desalted peptides were resuspended in 5 % ACN, 10 mM NH 4 HCO 3 pH 8 and fractionated in a basic pH reversed phase chromatography using a HPLC equipped with a 3.5 μm Zorbax 300 Extended-C18 column (Agilent). Fractions were collected in a 96-well plate, then combined into 24 samples. Twelve of them were desalted following the C18 Stop and Go Extraction Tip (STAGE-Tip) and dried down in a SpeedVac. Finally, peptides were resuspended in 1 % formic acid, 3 % ACN, and analyzed by LC-MS3 in an Orbitrap Fusion Lumos mounted with FAIMS and running in HR-MS^2^ mode[81].

#### 2.8.3. Proteomics – data analysis

A suite of in-house pipeline software (GFY-Core Version 3.8, Harvard University) was used to obtain final protein quantifications from all RAW files collected. RAW data were converted to mzXML format using a modified version of RawFileReader (5.0.7) and searched using the search engine Comet[82]against a human target-decoy protein database (downloaded from UniProt in June 2019) that included the most common contaminants. Precursor ion tolerance was set at 20 ppm and product ion tolerance at 0.02 Da. Cysteine carbamidomethylation (+57.0215 Da) and TMT tag (+229.1629 Da) on lysine residues and peptide N-termini were set as static modifications. Up to two variable methionine oxidations (+15.9949 Da) and two missed cleavages were allowed in the searches. Peptide-spectrum matches (PSMs) were adjusted to a 1 % FDR with a linear discriminant analysis[83] and proteins were further collapsed to a final protein-level FDR of 1 %. TMT quantitative values we obtained from MS2 scans. Only those with a signal-to-noise ratio >100 and an isolation specificity > 0.7 were used for quantification. Each TMT was normalized to the total signal in each column. Quantifications are represented as relative abundances. The mass spectrometry proteomics data have been deposited to the ProteomeXchange Consortium via the PRIDE[84] partner repository with the dataset identifier PXD056511 and 10.6019/PXD056511. Further details on the TMT method, instrument parameters, and sample information can be found in Supplementary Table 1. Enrichment of GO-terms (CC, Cellular Component and KEGG pathways) was performed using DAVID Functional Annotation Tool[85]. For these analyses, all proteins that were significantly (p-value>0.05) up-or downregulated between WT and treated cells were considered, without applying a specific threshold. The annotation list for the subcellular localization of organellar protein markers was derived from previously published high confidence HeLa dataset[86]; “high” and “very high” confidence. MitoCarta 3.0 [87]. was used for mitochondrial annotation. Figures were generated using a combination of Excel, Perseus (v1.6.5)[88], GraphPad Prism (v8.0), and SRplot (https://www.bioinformatics.com.cn/en). Supplementary Tables 2–3 list all quantified proteins as well as associated TMT reporter ratio to control channels used for quantitative analysis.

### 2.9. Microscopy

#### 2.9.1. Live-cell confocal microscopy for mitophagic flux analysis

For quantitative mtx-QC(mCherry-GFP)^XL^ flux analysis iNeurons were plated onto μ-Slide 8 well ibiTreat (Ibidi) on day 7 of differentiation. On day 11–12, the cells were treated with IU1–47 (5–10 μM) or Antimycin A (0.5 μM) and Oligomycin (0.5 μM) for 24 H. Cell were imaged using the laser spinning disk confocal iMIC-Andromeda imaging workstation (TILL Photonics, Graefelfing, Germany) equipped with UPlanSApo 60X/1.35 objective lens. Images for mCherry and eGFP were collected sequentially using 561 nm and 488 nm solid state lasers and emission collected with 615/20 and 525/39 filters, respectively. Consistent laser intensity and exposure time were applied to all the samples, and brightness and contrast were adjusted equally by applying the same minimum and maximum display values in FiJi software[76].

Image Quantitation: For each condition a minimum of 10 image sections were taken with a 60x objective lens and analyzed using Fiji software[76]. All the sections were included in the analysis except the cells that showed lower GFP-mCherry expression levels compared to the average fluorescent intensity. Step 1) Following z-projection stack and background subtraction, a threshold (Otsu) was applied for each channel to create two binary images (green mask and red mask). Step 2) Binary images were subtracted (red mask – green mask) resulting in a binary image of “red only puncta” representing the mitolysosomes. The “Analyze Particles…” command (pixel size exclusion: 0.2-exclude edge objects) was used to measure the total puncta number puncta and mean area for each image. The number of cells present in each image was counted manually. Step 3) Mitophagy index was calculated for each image applying the following equation: [(n° of mitolysosome/n° of cells) × mean area of mitolysosomes]. The average value for each replicate in each condition was normalized by the average value obtained from replicates of the untreated condition.

#### 2.9.2. Mitophagic flux analysis in flies neurons

Flies were raised under standard conditions at 23°C with a 12:12 h light:dark cycle (unless differently stated), on agar, cornmeal, yeast food. Wild type (w^1118^) and driver lines nSyb-GAL4 (BDSC_51635) were obtained from Bloomington Drosophila Stock Center. The UAS-Usp14 RNAi line (KK-110227) was obtained from VDRC Stock Center. The line UAS-mito-QC was generated previously[89]. For larval experiments, L3 wandering larvae were selected based on their phenotypes. Larval brain dissection was performed in PBS and fixed in 4 % formaldehyde, pH 7.0 for 20 min. Subsequently, brains were washed in PBS and mounted on coverslips. Fluorescence microscopy imaging was performed using a Zeiss LSM 900 confocal microscope equipped with 100× Plan Apochromat (oil immersion, NA 1.4) objective lenses at 2× digital zoom. Z-stacks were acquired at 0.5 μm steps. For each larval brain, two images of different areas were taken. In the graphs, each data point represents one brain. For mitophagy analysis, samples were imaged via sequential excitations (488 nm, green; 561 nm, red). Laser power and gain settings were adjusted depending on the fluorophore but were maintained across samples. For mitolysosome quantification, the number of mCherry-only puncta was quantified using the mQC-counter plugin[90], maintaining the same parameters across samples.

#### 2.9.3. Immunocytochemical analysis

hESCs or iNeurons were plated on 13 mm round glass coverslips. For membrane potential assessment the cells were treated with IU1–47 (5–10 μM) or Antimycin A (0.5 μM) and Oligomycin (0.5 μM) for 24 H on day 11 and stained with 50 nM MitoTracker RED CMX Ros for 30 minutes before fixation. The iNeurons were fixed in 4 % PFA in PBS for 15 min at room temperature, permeabilized with 0.1 % Triton X-100 in 1xPBS/0.05 % Tween20 for 15 min at RT and blocked for 1 hour at RT in 4 % BSA in 1xPBS/0.05 % Tween20. Anti-TOM20 antibody was diluted at 1:200 in 1xPBS/0.05 % Tween20 and 1 % BSA and applied overnight at 4°C. Secondary antibodies were diluted at 1:400 in 1xPBS/0.05 % Tween20 and 1 % BSA and applied for 1 h at room temperature. Coverslips were mounted on cover slides using Moviol mounting medium.

Cells were imaged using the laser spinning disk confocal iMIC-Andromeda imaging workstation (TILL Photonics, Graefelfing, Germany) equipped with UPlanSApo 60X/1.35 objective lens. Images were collected using 561 nm and 488 nm solid state lasers and emission collected with 615/20 and 525/39 filters, respectively according to the secondary antibody used in the experiment. Consistent laser intensity and exposure times were applied to all samples, and brightness and contrast were adjusted equally by applying the same minimum and maximum display values in FiJi software[76].

#### 2.9.4. Transmission electron microscopy

Samples were fixed with 2.5 % glutaraldehyde in 0.1 M sodium cacodylate buffer pH 7.4 ON at 4°C. The samples were postfixed with 1 % osmium tetroxide plus potassium ferrocyanide 1 % in 0.1 M sodium cacodylate buffer for 1 hour at 4°C. After three water washes, samples were dehydrated in a graded ethanol series and embedded in an epoxy resin. Ultrathin sections (60–70 nm) were obtained with an Ultrotome V (LKB) ultramicrotome, counterstained with uranyl acetate and lead citrate, and viewed with a Tecnai G2 (FEI) transmission electron microscope operating at 100 kV. Images were captured with a Veleta (Olympus Soft Imaging System) digital camera.

### 2.10. Statistical analysis

Data are presented as mean ± SEM from at least three independent experiments. The exact number of replicates (N) for each experiment is indicated in the figure legend. Statistical significance was determined using unpaired t-test, or multiple comparison test (One-way ANOVA) with relevant post-hoc test, and p-values are indicated. Details on the statistical test performed and p-values are specified in every figure legend. Statistical significance is identified as follow: *=p-value ≤ 0,05; **= p-value ≤ 0,01; ***= p-value ≤ 0,001(GraphPad Prism 8 software).

## 3. Results

### 3.1. In iNeurons USP14 inhibition induces differential remodelling of the mitochondrial proteome

To dissect the molecular pathway underlying the effect of USP14 inhibition elicited in previous works[55,59,68,69,91], and to identify the repertoire of USP14 substrates that accounts for its protective effects, we performed a mass spectrometry-based analysis of iNeurons in which USP14 activity was inhibited. We first performed a dose-response MTT assay to evaluate the potential toxicity of IU1–47, a potent and highly selective inhibitor of USP14[59]. We treated iNeurons with increasing dosages (1–200 μM) of IU1–47 for 24 H, and plotted cell survival. We found that at doses up to 10 μM, at least 80 % of viability was retained (Figure S1A). The effect was reduced in USP14 KO cells as expected, while at higher concentrations (>50 μM) we detected some level of toxicity in iNeurons (Figure S1A) as well as in SH-SY5Y cells (Figure S1B). These results support previously reported evidence on the relative lack of toxicity of IU1–47 in cells[59].

**Fig. 1.**
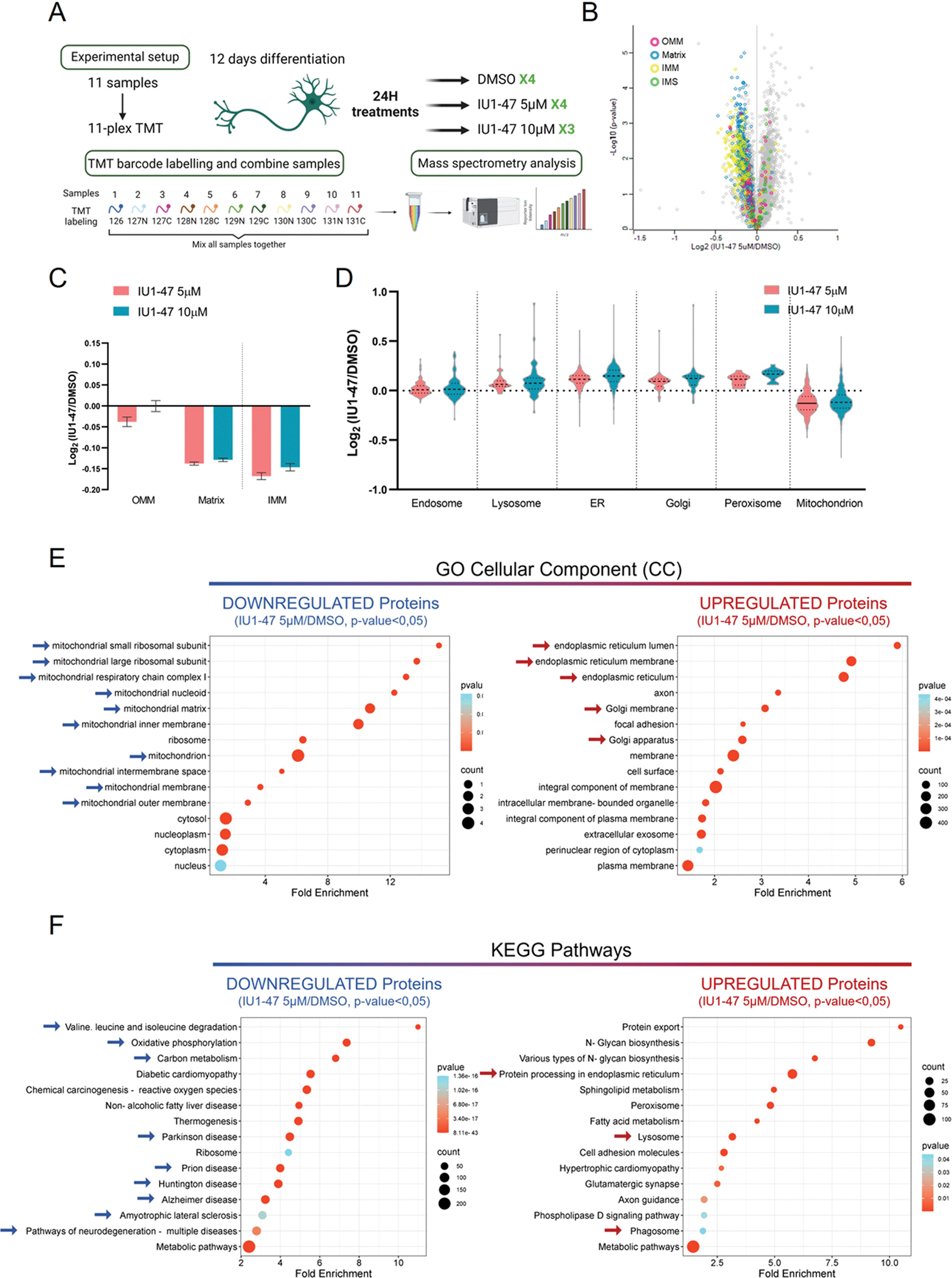
TMT-analysis of iNeurons with USP14 inhibition. (A) Workflow for TMT-based proteomics of iNeurons. 11-plex proteomics was performed on 4 biological replicates for Control (DMSO) and IU1–47–5μM treatments, and 3 biological replicates for IU1–47–10μM treatments. (B) Volcano plots representing the abundance of the 8018 identified proteins in the WT iNeurons treated with 5 μM IU1–47 for 24 H compared with untreated cells (DMSO). Mitochondrial proteins (identified by comparison with MitoCarta 3.0) are represented with colored dots based on their reported mitochondria localization: OMM proteins (magenta), matrix proteins (blue), IMM proteins (yellow), IMS proteins (green). (C) Distribution of changes in protein abundance for proteins that localize in the mitochondria matrix, the IMM, or the OMM in iNeurons treated with 5 μM (pink) or 10 μM (blue) IU1–47 for 24 H compared with untreated cells (DMSO). (D) Distribution of changes in protein abundance for proteins that localize in individual organelles or protein complexes in iNeurons treated with 5 μM (pink) or 10 μM (blue) IU1–47 for 24 H compared with untreated cells (DMSO). (E) GO-term Cellular Component analysis of proteins significantly downregulated (left panel) (p-value<0.05) or upregulated (right panel) in iNeurons treated with 5 μM IU1–47–24H compared to control (DMSO). (F) KEGG pathways enrichment analysis of proteins significantly downregulated (left panel) (p-value<0.05) or upregulated (right panel) in iNeurons treated with 5 μM IU1–47–24H compared to control (DMSO). Blue arrows identify particularly interesting pathways.

We treated WT iNeurons with sub-toxic concentrations of IU1–47 (5 μM and 10 μM) for 24 H, and compared them with untreated samples (DMSO). Sample treatments were performed in quadruplicate (DMSO and 5 μM IU1–47) or triplicate (10 μM IU1–47), and total cell extracts were subjected to 11-plex Tandem Mass Tagging (TMT)-based proteomics (Fig. 1A). Replicates were highly correlated, and Principal Component Analysis (PCA) revealed clustering of replicates, with PC1 clearly separating treated samples (IU1–47) from controls (DMSO) (Figure S1C). Since only one of the CTR samples was separated from the others in PC2 (DMSO_4), we excluded this sample from the subsequent analysis. TMT proteomics quantified 8018 proteins, and through annotation of mitochondrial proteins using the MitoCarta 3.0 database, we found major alterations in the abundance of the mitochondrial proteome following USP14 inhibition with both 5 μM(Fig. 1B) and 10 μM (Figure S1D) IU1–47 treatment.

The majority of proteins annotated as mitochondrial were downregulated in IU1–47-treated iNeurons compared to CTR, as indicated in the volcano plots (leftward skew of coloured dot in Fig. 1B and Figure S1D). Proteins with decreased abundance were enriched for IMM, Matrix, and to a less extent, OMM sub-organelle compartment categories (Fig. 1C). Importantly, other organelles were not negatively affected by IU1–47 treatment; in fact, we found mild increases in proteins belonging to the ER, Golgi, and Peroxisome, together with an upregulation of the lysosomal compartment (Fig. 1D).

We next performed Gene Ontology analysis on the subset of data obtained from the treatment with 5 μM of IU1–47 compared to CTR, and confirmed the specific enrichment of mitochondrial proteins in the downregulated subset (Fig. 1E; left panel), while the upregulated subset was enriched in proteins belonging to ER and Golgi (Fig. 1E; right panel). On the same dataset, we also performed KEGG pathway enrichment analysis, which highlighted a downregulation of pathways involved in mitochondrial functions as well as different neurodegenerative diseases, including PD, AD, and ALS (Fig. 1F; left panel). KEGG Pathway enrichment also showed upregulation of “lysosome”, “phagosome”, and “protein processing in the ER” pathways (Fig. 1F; right panel), supporting the hypothesis that the autophagy-lysosomal pathway is activated in this condition. Similar results were obtained in comparing WT and USP14 KO iNeurons using the same TMT-based approach; in this case, the downregulation of mitochondrial proteins appeared to be less pronounced (Figure S1E–G).

The results above indicate that in iNeurons USP14 inhibition specifically induces downregulation of the mitochondrial proteome, without impacting other cellular organelles.

### 3.2. Inhibition of USP14 induces autophagy in iNeurons

Considering the correlation between USP14 and autophagy highlighted in previous works[56,58,68], and the identification in our TMT analysis of specific downregulation of the mitochondrial proteome and the upregulation of autophagy-lysosomal pathway, we next wanted to evaluate autophagy levels in iNeurons when USP14 activity is inhibited.

**Fig. 2.**
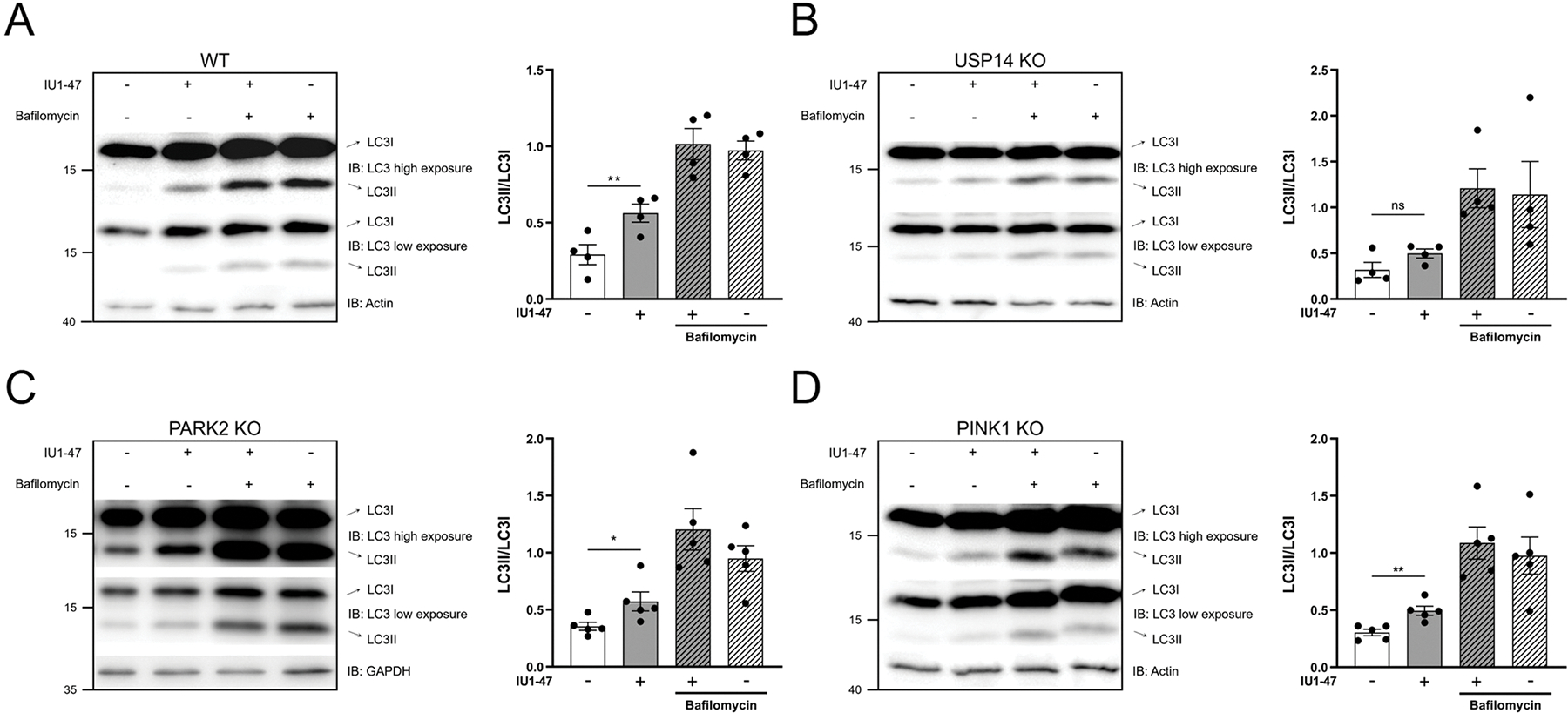
Autophagy mediated by USP14 inhibition. (A-B) Western Blot analysis of the indicated proteins, and corresponding quantifications in WT (A) and USP14 KO (B) iNeurons treated with 10 μM IU1–47 for 24 H. Treated samples display a significant increase of LC3II:LC3I ratio compared to control in WT cells but not in USP14 KO indicating specificity of the inhibition. Treatment with Bafilomycin A1 (10 nM) alone, and in combination with IU1–47 was used as control to inhibit the autophagic flux. Graph bar represent mean±SEM. N=4 independent experiments. (C-D) Western Blot analysis of the indicated proteins, and corresponding quantifications in PARK2 KO (C) and PINK1 KO (D) iNeurons treated with 10 μM IU1–47 for 24 H. Treatment with Bafilomycin A1 (10 nM) alone, and in combination with IU1–47 was used as control. N≥4 independent experiments. Graph bar represent Mean±SEM. Statistical significance determined by one-way ANOVA with Bonferroni post-correction test. *=p-value ≤ 0,05; **= p-value ≤ 0,01; ***= p-value ≤ 0001.

We assessed autophagy by western blotting analysis of LC3 levels in two separate *in vitro* models, iNeurons and SH-SY5Y neuroblastoma cells. In WT iNeurons, USP14 inhibition by IU1–47 (10μM-24H) induced an increase of autophagy, represented by an increased ratio between the lipidated (LC3II) and unmodified (LC3I) forms of LC3 protein (Fig. 2A). We observed a further increase in LC3II levels when cells were co-incubated with IU1–47 and bafilomycin (10nM-24H) (Fig. 2A). Importantly, IU1–47 did not seem to affect the LC3II:LC3I ratio in USP14 KO cells (Fig. 2B). A similar result was obtained in SH-SY5Y cells treated with a sub-toxic concentration of IU1–47 (Figure S2A–B). Inhibition of USP14 by IU1–47 enhanced autophagy also in PARK2 KO (Fig. 2C) and PINK1 KO (Fig. 2D) iNeurons, demonstrating that the autophagic effect of USP14 inhibition is PINK1/Parkin-independent.

We obtained similar results with the less potent USP14 inhibitor, IU1 (100 μM/24–48hrs), for which we observed a significant increase in the LC3II:LC3I ratio, both in WT and PINK1 KO iNeurons (Figure S3A). Transmission Electron microscopy (TEM) analyses revealed a significantly increased number of autophagic vesicles after IU1 treatment in both genotypes (Figure S3B). We detected no differences in the number of mitochondria, while the mitochondrial area was found significantly smaller in WT iNeurons treated with IU1 for 48hrs (Figure S3B). Less potent inhibitor IU1 seemed to display off-target effects because the autophagic response triggered by IU1 was not completely abrogated in USP14 KO background (Figure S3C–D).

In summary, USP14 inhibition by IU1–47 enhances autophagy in iNeurons with a mechanism that is PINK1/Parkin independent.

### 3.3. Inhibition of USP14 induces mitophagy in iNeurons in a PINK1/Parkin-independent fashion

**Fig. 3.**
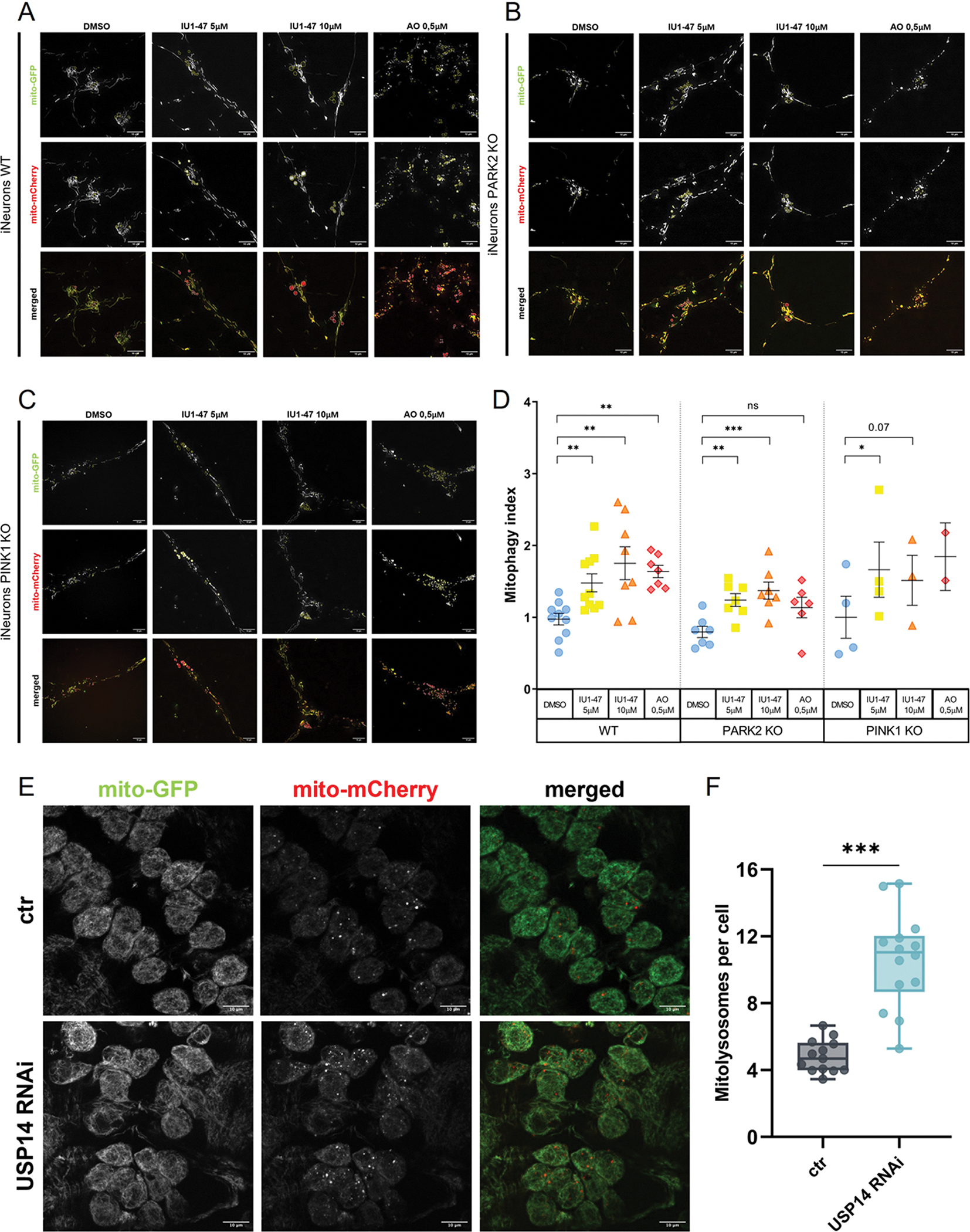
Mitophagy mediated by USP14 inhibition. (A-C) Representative images of WT (A), PARK2 KO (B) and PINK1 KO (C) iNeurons expressing mtx-QCXL treated with 5 μM and 10 μM IU1–47 for 24 H or depolarized with 0.5 μM antimycin A/0.5 μM oligomycin (sub-threshold depolarization) for 24 H, and imaged for mCherry (red) and GFP (green). Cells were imaged as described in Material and Methods. (D) Quantification of the mitophagic flux in WT, PARK2 KO and PINK1 KO iNeurons (as described in Material and Methods) treated with 5 μM and 10 μM IU1–47 for 24 H or depolarized with 0.5 μM antimycin A/0.5 μM oligomycin (sub-threshold depolarization) for 24 H. Dots represent biological replicates, for each replicate N≥10 images per treatment were analyzed. Error bars represent Mean±SEM. Statistical significance determined by one-way ANOVA with Dunnett’s post-correction test. *=p-value ≤ 0,05; **= p-value ≤ 0,01; ***= p-value ≤ 0001. (E) Confocal microscopy analysis of larval VNC neurons expressing mito-QC. mCherry puncta represent mitolysosomes under basal conditions (ctr) or upon USP14 genetic down-regulation (USP8 RNAi) (**F**) Quantification of mitolysosomes per cell in the two different conditions. Statistical significance determined by one-way ANOVA with Dunnett’s post-correction test. *=p-value ≤ 0,05; **= p-value ≤ 0,01; ***= p-value ≤ 0001.

Our results support the hypothesis of a proteostatic effect of USP14 inhibition in iNeurons, which specifically affects the mitochondrial proteome (Fig. 1), and involves the activation of autophagy (Fig. 2). Based on these results, the logical question was whether USP14 inhibition affected mitophagy. To evaluate basal mitophagy in iNeurons, we took advantage of the mitophagy flux reporter developed by Ordureau et al., called mtx-QC^XL^[72]. mtx-QC^XL^ is a mCherry-GFP tandem protein that is targeted to the mitochondrial matrix, and allows monitoring ongoing mitophagy by fluorescent microscopy. Mitochondria are stained as red-green entities under normal conditions. Delivery of mtx-QC^XL^ to the acidic environment of the lysosomes results in GFP quenching, and selective accumulation of mCherry-positive mitochondria [72]. This construct was stably introduced into engineered hESCs (WT, PARK2 KO and PINK1 KO). We differentiated hESCs of the indicated genotype (WT, PARK2 KO and PINK1 KO) into iNeurons, and we treated them with IU1–47 (5–10 μM – 24 H). As a positive control for mitophagy induction, iNeurons were treated with 0.5 μM antimycinA/0.5 μM oligomycin for 24 H (AO, sub-threshold depolarization). Mitophagy flux was assessed using live-cell imaging, evaluating the presence of mCherry-positive puncta in mtx-QC^XL^. WT iNeurons displayed a significant and concentration-dependent increase of the mitophagic flux upon treatment with IU1–47; this increase was comparable with the one obtained upon stress-induced mitophagy with AO (Fig. 3A, quantified in 3D). In PARK2 KO iNeurons, the mitophagic effect of USP14 inhibition (5–10 μM IU1–47/24H) was still readable, while stress-induced mitophagy triggered by AO was significantly reduced (Fig. 3B, quantified in 3D). Similar results were obtained in the PINK1 KO background (Fig. 3C, quantified in 3D). In parallel to *in vitro* studies, we also monitored the mitophagic flux induced by USP14 inhibition in an *in vivo* genetic model of Drosophila melanogaster, in which USP14 was downregulated by RNAi. We measured mitophagy in the Drosophila brain by expressing the mitophagic fluorescent reporter probe mito-QC in the neurons of the ventral nerve chord (VNC) of third instar stage larvae. In WT flies, we observed on average five mitolysosomes per cell, whereas in the brains of USP14-down-regulating flies, data analysis showed a significant increase in the number of mitolysosomes, indicative of enhanced mitophagic flux in this condition (Fig. 3E–F).

In summary, USP14 inhibition enhances basal mitophagy in human neurons, and in the Drosophila brain. The mitophagic effect of USP14 inhibition is PINK1/Parkin-independent.

### 3.4. IU1–47-induced mitophagy is MARCH5-dependent

Our data indicate the existence of a mitophagic pathway that is activated by USP14 inhibition, and does not operate via the canonical PINK1/Parkin pathway. To explore the molecular mechanism leading to the mitophagic effect of USP14 inhibition, we took advantage of different hESCs cell lines lacking specific mitophagy receptors and/or regulators, namely BNIP3L/NIX KO, MUL1 KO, and MARCH5 KO. BNIP3L/NIX is an autophagic receptor that localizes on mitochondria. It is a key regulator of PINK1/Parkin -independent mitophagy induced by iron chelation (DFP) hypoxia[92] and organelle remodeling during differentiation [72]. MUL1 is a multifunctional mitochondrial membrane protein that acts as an E3 ubiquitin ligase that binds, ubiquitinates, and degrades Mfn2[93], and as a SUMO E3 ligase towards Drp1 to regulate mitochondrial fission[94]. MUL1 can also regulate Parkin-independent mitophagy via an unknown mechanism[95,96], and it acts as an early checkpoint to suppress neuronal mitophagy under mild stress, by degrading Mfn2 and enhancing ER-Mito coupling[97]. Finally, MARCH5 (also named MITOL) is a mitochondrially localized RING finger E3 ubiquitin ligase involved in mitochondrial dynamics, ubiquitinating multiple mitochondrial substrates such as Fis1, Mfn1, Mfn2, and MiD49 (reviewed by Shiiba et al.[98]). MARCH5 regulates hypoxia-induced mitophagy through ubiquitination of mitophagy receptor FUNDC1[99], thus placing it at the crossroads between regulation of mitochondrial dynamics and quality control.

**Fig. 4.**
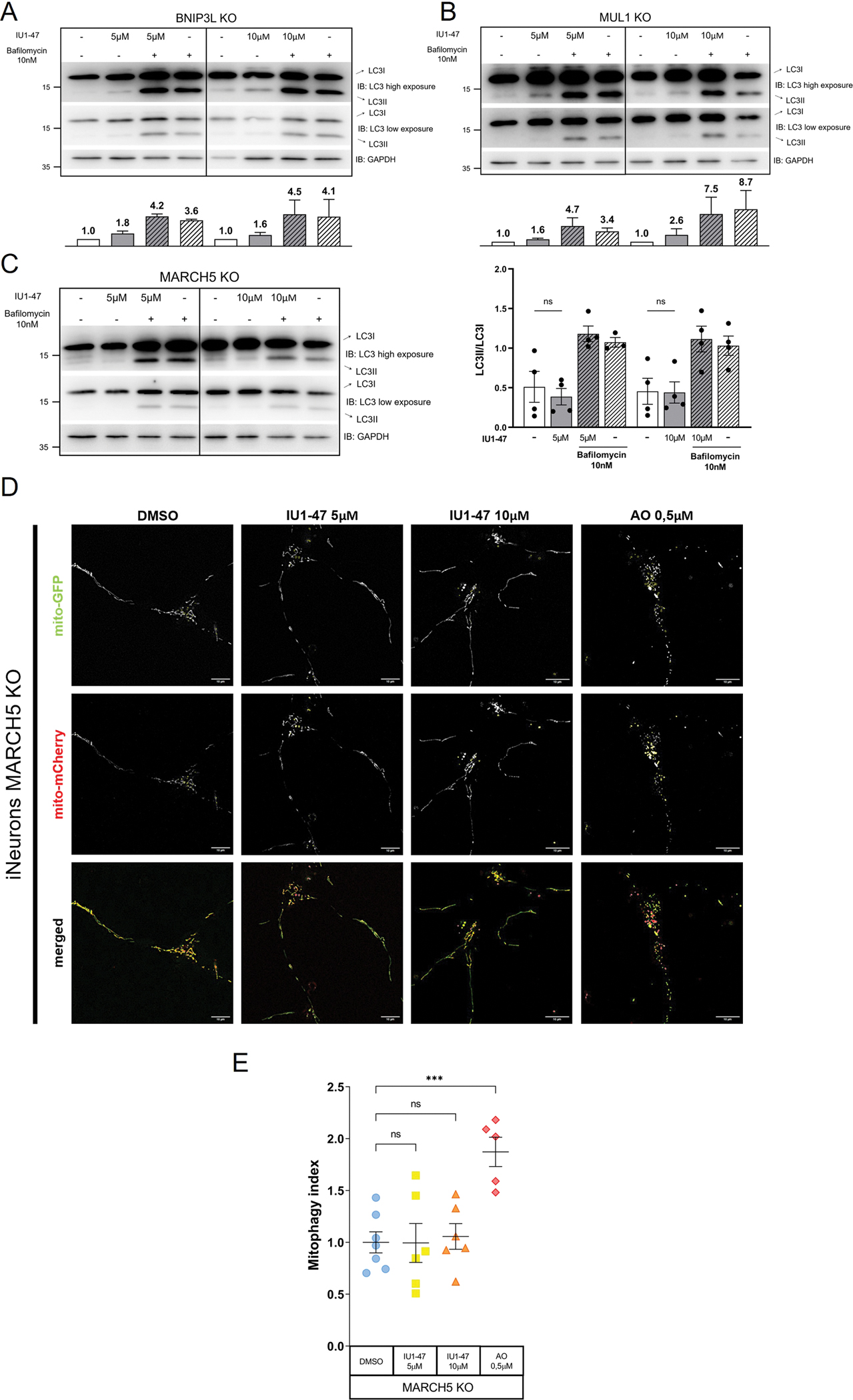
USP14-mediated autophagy is MARCH5-dependent. (A-B) Western Blot analysis of the indicated proteins, and corresponding quantification compared to untreated sample (graph bar below) in (A) BNIP3L KO and (B) MUL1 KO iNeurons treated with 5 and 10 μM IU1–47 for 24 H. Treatment with Bafilomycin A1 (10 nM) alone, and in combination with IU1–47 was used as control. N=2 independent experiments. Graph bar represent Mean±SD. (C) Western Blot analysis of the indicated proteins and corresponding quantification compared to untreated sample in MARCH5 KO iNeurons treated with 5 and 10 μM IU1–47 for 24 H. Treatment with Bafilomycin A1 (10 nM) alone, and in combination with IU1–47 was used as control. N=4 independent experiments. Graph bar represent Mean±SEM. Statistical significance determined by one-way ANOVA with Bonferroni post-correction test. *=p-value ≤ 0,05; **= p-value ≤ 0,01; ***= p-value ≤ 0001. (D) Representative images of MARCH5 KO iNeurons expressing mtx-QCXL treated with 5 μM and 10 μM IU1–47 for 24 H or depolarized with 0.5 μM antimycin A/0.5 μM oligomycin (sub-threshold depolarization) for 24 H, and imaged for mCherry (red) and GFP (green). (E) Quantification of the mitophagic flux in MARCH5 KO iNeurons treated with 5 μM and 10 μM IU1–47 for 24 H or depolarized with 0.5 μM antimycin A/0.5 μM oligomycin (sub-threshold depolarization) for 24 H. Dots represent biological replicates, for each replicate N≥10 images per treatment were analyzed. Error bars represent Mean±SEM. Statistical significance determined by one-way ANOVA with Dunnett’s post-correction test. *=p-value ≤ 0,05; **= p-value ≤ 0,01; ***= p-value ≤ 0001.

We differentiated iNeurons from BNIP3L/NIX KO, MUL1 KO and MARCH5 KO hESCs, treated them with IU1–47 (5–10 μM – 24 H), and first evaluated autophagy. As before, we used bafilomycin (10nM-24H) to monitor the autophagic flux. We found that IU1–47 induces autophagy in BNIP3L/NIX KO (Fig. 4A) and MUL1 KO neurons (Fig. 4B), but not in the MARCH5 KO background (Fig. 4C). We next measured mitophagy in MARCH5 KO iNeurons treated with IU1–47, and found that mitophagy induction was abolished in MARCH5-deficient cells (Fig. 4D–E).

Thus, the mitophagic effect of USP14 inhibition is MARCH5-dependent.

### 3.5. USP14 inhibition stimulates mitochondrial biogenesis

**Fig. 5.**
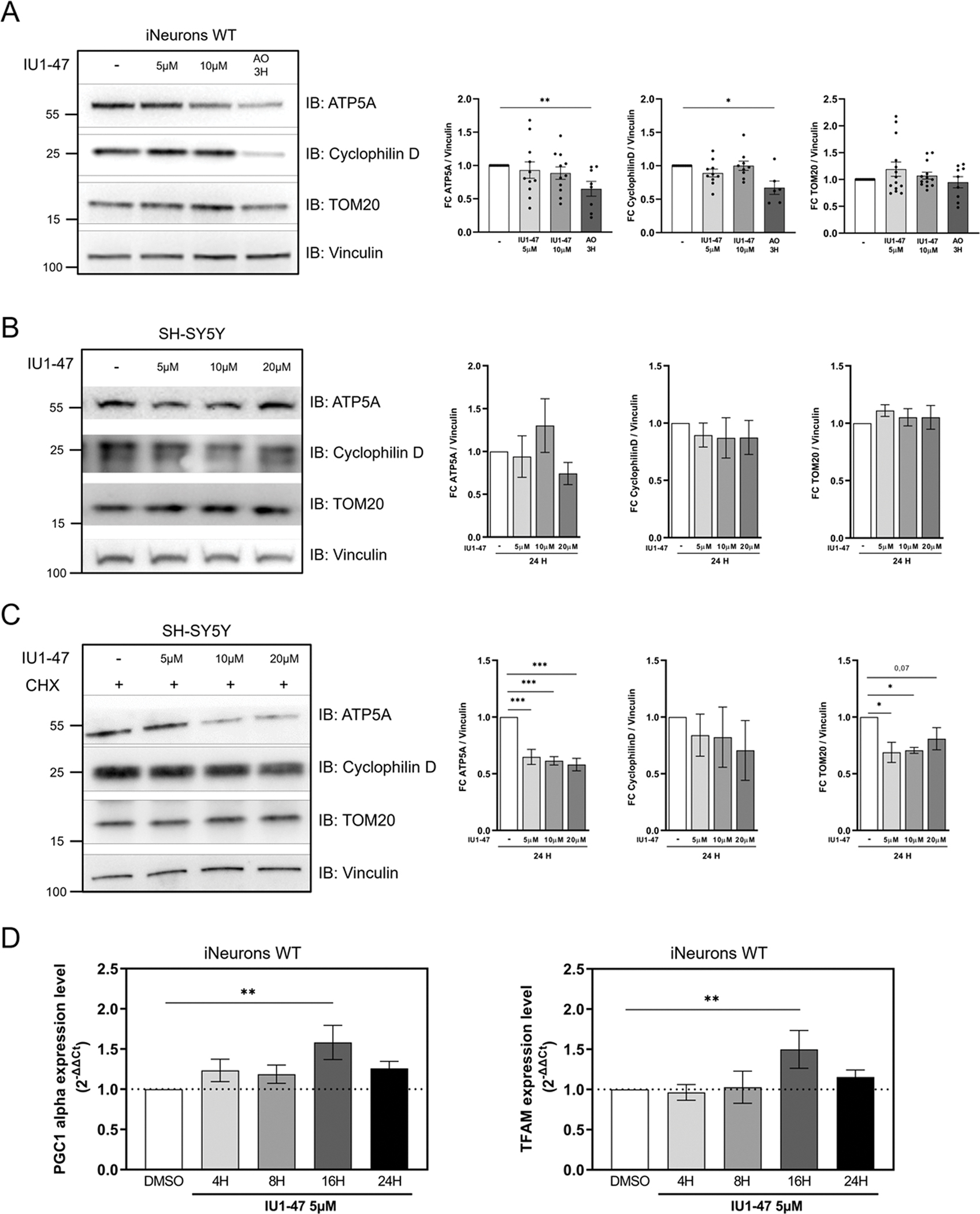
Inhibition of USP14 stimulates mitochondrial biogenesis. (A) Western Blot analysis of the indicated proteins in WT iNeurons treated with 5 μM and 10 μM IU1–47 for 24 H or depolarized with 10 μM antimycin A/5 μM oligomycin for 2 H. Charts show mean ± SEM of n=9 biological replicates. (B) SH-SY5Y cells were treated with different concentrations of IU1–47 for 24 H and protein levels of ATP5A, TOM20 and CyclophilinD were assessed by Western Blot analysis. Charts show mean ± SEM of n=3 biological replicates. (C) SH-SY5Y cells were treated with 10 μg/ml of CHX for 24 H in combination with different concentrations of IU1–47 for 24 H. Levels of the indicated proteins were assessed by Western Blot analysis. Charts show mean ± SEM of n=3 biological replicates. In all conditions, Vinculin was used as loading control. Data are represented as fold change compared to control, and DMSO was used as control. Statistical significance determined by one-way ANOVA with Dunnett’s post-correction test. *=p-value ≤ 0,05; **= p-value ≤ 0,01; ***= p-value ≤ 0001. (D) Real Time qPCR analysis of PGC-1α and TFAM transcription levels on WT iNeurons treated with 5 μM IU1–47 for the indicated time. N=10 independent experiments. Graph bars represent Mean±SEM. Statistical significance determined by one-way ANOVA with Dunnett’s post-correction test. *=p-value ≤ 0,05; **= p-value ≤ 0,01; ***= p-value ≤ 0001.

If mitochondria are degraded, and this is not compensated by mitochondrial biogenesis, mitochondrial loss should be reflected by a significant decrease in the levels of mitochondrial mass. However, we did not observe a significant decrease in the levels of mitochondrial resident proteins Cyclophilin D (mitochondrial matrix), ATP5A (inner mitochondrial membrane), and TOM20 (outer mitochondrial membrane) in iNeurons (Fig. 5A), nor in SH-SY5Y cells (Fig. 5B), unless protein synthesis was inhibited by cycloheximide (Fig. 5C). Thus, we next addressed whether mitophagy induction by USP14 inhibition was paralleled by the simultaneous activation of mitochondrial biogenesis. We first evaluated mtDNA copy number, and observed a significant increase in mtDNA copy number in iNeurons treated with IU1–47 (Figure S4A). Next, we assessed mitochondrial biogenesis by evaluating transcript levels of mitochondrial biogenesis transcription co-activator PGC1alpha[100,101] and mitochondrial transcription factor A (TFAM) [102]. Transcript levels of PGC1alpha and TFAM were upregulated in iNeurons (Fig. 5D) upon IU1–47 treatment. We also examined transcriptional levels of PGC1alpha and TFAM in PARK2 KO iNeurons treated with IU1–47. It was interesting to observe that in this condition, the effect on mitochondrial biogenesis induced by IU1–47 was abrogated (Figure S4B–C).

In summary, inhibition of USP14 enhances overall mitochondrial turnover by stimulating both mitochondrial biogenesis and mitophagy.

### 3.6. USP14 inhibition rescues mitochondrial respiratory defects of PARK2 KO iNeurons

**Fig. 6.**
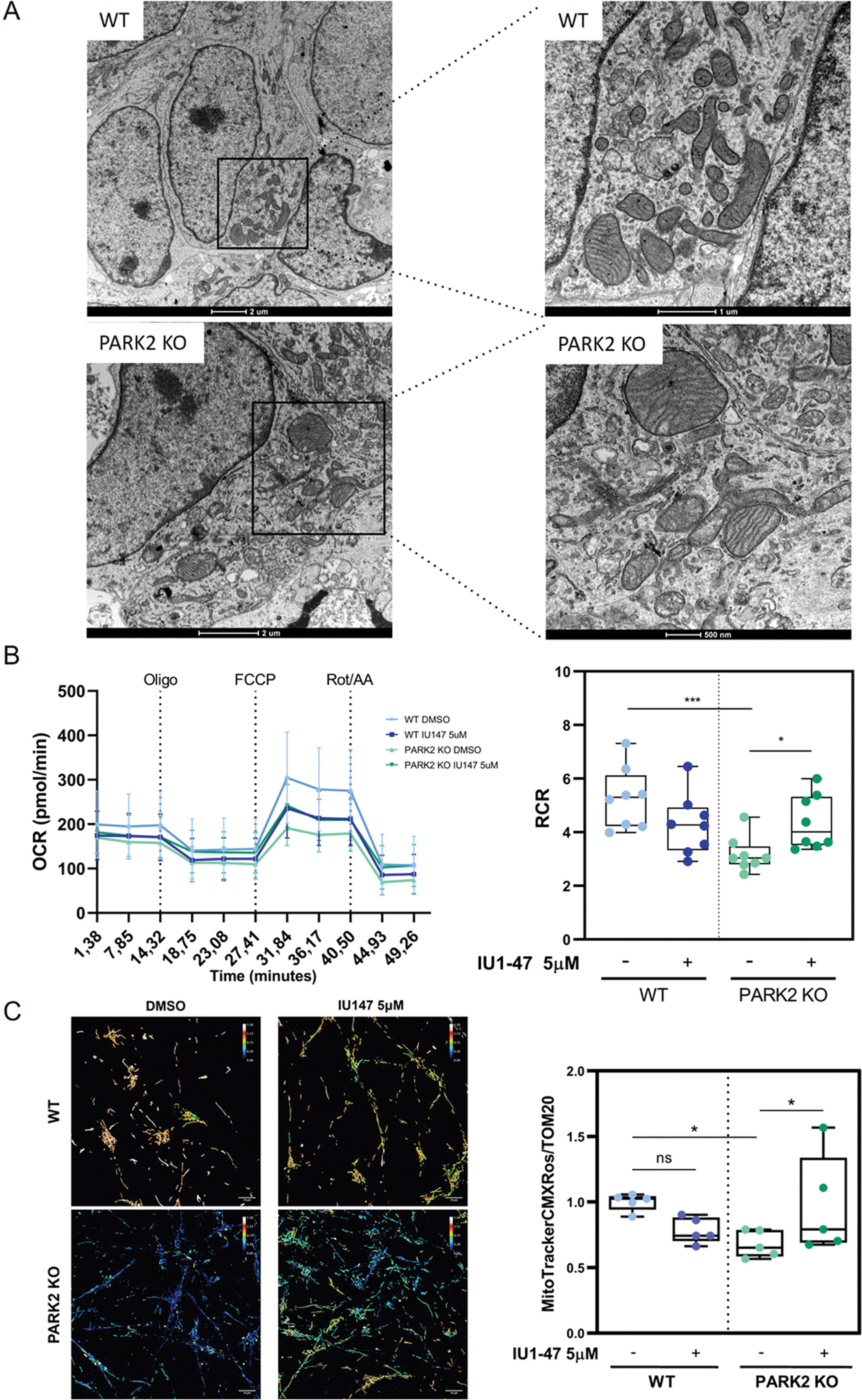
Enhanced mitophagy rescues mitochondrial respiratory defects of PARK2 KO iNeurons. (A) Representative transmission electron microscopy (TEM) images of iNeurons of the indicated genotypes (WT, PARK2 KO). Enlarged images are illustrated on the right panel showing details of mitochondrial ultrastructure and shape. In Parkin KO background, swollen and ultrastructurally deranged mitochondria are visible. (B) (Left) Plot showing mitochondrial oxygen consumption rate measurements (OCR) of WT and PARK2 KO iNeurons treated with 5 μM IU1–47 for 24 H. OCR measurements were performed using the Seahorse XFe24 using the indicated treatments. (Right) Corresponding quantification of respiratory control ratio (RCR) calculated as State 3/State 4. N=8 independent experiments represented by dots. Ordinary one-way ANOVA with Holm-Sidak’s multiple comparisons test was used to compare the RCR between genotypes and treatments. *=p-value ≤ 0,05; **= p-value ≤ 0,01; ***= p-value ≤ 0001 (C) (Left) Immunofluorescence staining of mitochondrial marker Tom20 and MitoTracker Red CMXRos on WT and PARK2 KO iNeurons treated with 5 μM IU1–47 for 24 H. Images represent Fluorescence ratio (MitoTracker Red CMXRos/Tom20) between the two probes with representative colors depicted in the colored scale bar. (Right) Corresponding quantification of MitoTracker Red CMXRos/Tom20 intensity ratio. N=5 independent experiments represented by dots. Ordinary one-way ANOVA with Holm-Sidak’s multiple comparisons test was used to analyse differences between genotypes and treatments. *=p-value ≤ 0,05; **= p-value ≤ 0,01; ***= p-value ≤ 0001.

The observation that USP14 inhibition promotes basal mitochondrial turnover in iNeurons and in SH-SY5Y cells points to a potential beneficial effect of USP14 inhibition in models in which accumulation of dysfunctional mitochondria is implicated. Thus, after establishing that we can induce PINK1/Parkin-independent mitophagy and mitochondrial biogenesis through USP14 inhibition, we sought to understand if this is beneficial for PARK2 KO neurons, which develop mitochondrial dysfunction[50,103]. Parkin KO iNeurons show a clear mitochondrial-related phenotype with swollen mitochondria and misarranged mitochondrial cristae structure, and a decreased number of electron-dense mitochondria (Fig. 6A). We measured mitochondrial respiration in WT and PARK2 KO iNeurons using Seahorse XF24 Flux Analyzer (Agilent Technologies, USA), and we found a significant reduction in the Respiratory Control Ratio (RCR) of Parkin-deficient neurons, supporting the results of Kumar et al. in DA neurons[103]. Treatment with IU1–47 (5μM– 24 H) rescued the impaired mitochondrial phenotype of PARK2 KO iNeurons, while it did not have a significant impact on the respiration of WT cells (Fig. 6B).

To consolidate this finding, we also evaluated mitochondrial membrane potential using MitoCMXRos in conjunction with TOMM20 immunostaining. We found a 32 % reduction in mitochondrial membrane potential in PARK2 KO iNeurons compared to WT that was completely recovered upon IU1–47 (5 μM – 24 H) (Fig. 6C). We also measured mitochondrial respiration in PINK1 KO iNeurons, but did not record any significant impairment in this genotype compared to WT cells (Figure S5A).

In conclusion, mitochondrial defects of Parkin KO iNeurons can be rescued by inhibition of USP14.

## 4. Discussion

Mitochondrial function is central to cellular metabolism, apoptosis, and inflammation[104]. Thus, loss of mitochondrial homeostasis, and the accumulation of dysfunctional mitochondria are emerging as important hallmarks in diverse pathological conditions, including neurodegenerative disorders[22]. The maintenance of mitochondrial homeostasis is tightly regulated, and controlled by a series of interconnected pathways, defined as mitochondrial quality control (MQC), able to survey and preserve a functional mitochondrial repertoire by balancing mitochondria biogenesis and their degradation. At the molecular level, proteolysis activated by the mitochondrial proteases (the so-called mitochondrial unfolded protein response-mtUPR), and consequent proteasome-dependent degradation, can selectively remove damaged and misfolded proteins in mitochondria[105]. At the organelle level, the damage can be repaired by mitochondrial fission and fusion, which allow functional complementation of the damage within the mitochondrial network (fusion), while promoting its segregation through asymmetric mitochondrial fission[106,107]. Alternatively, upon mild damage generating oxidized proteins, mitochondrial particles accumulating damaged proteins can be delivered to lysosomes by the mitochondria-derived vesicle (MDV) pathway[108–111]. If mitochondria are severely damaged, the entire organelle can be delivered for degradation via mitophagy[36,112]. Ultimately, extensive and irreversible mitochondrial damage can lead to apoptosis[113].

In these degradative processes, the activation of the ubiquitin proteasome system (UPS) is crucial, and therefore regulators of the ubiquitination are attractive candidates for the development of drugs targeting mitochondrial turnover in neurodegenerative conditions [114]. Among regulators of ubiquitination, deubiquitinating enzymes (DUBs) are particularly attractive for their capacity to fine-tune the ubiquitination status of their targets through the removal of ubiquitin chains[115]. Recent studies have identified several DUBs involved in the modulation of ubiquitin-dependent mitochondrial turnover, such as ataxin-3[116,117], USP14[68], USP15[118], USP30[71], USP35[119] and USP8[73,120] (reviewed by Burtscher et al.[114], Chakraborty et al.[115], and Nardin et al.[121]). Among these enzymes, the proteasome-associated DUB USP14 is a particularly appealing target of inhibition because of its capacity to both enhance the activity of the UPS [54,55] and autophagy[56–58], and for its rescue effect against accumulation of intracellular proteotoxic protein aggregates[54,61–64,66]. More recently, selective inhibition of USP14 proved to be protective in PINK1/Parkin KO flies modeling motor and non-motor phenotype of PD [68,69].

Studies directed at the restoration of mitochondrial turnover in human neurons, and its potential protective effect in models of neurodegeneration are still scarce; for this reason, we evaluated the effect of USP14 inhibition in a human–derived embryonic stem cell line that is able to rapidly differentiate into functional iNeurons[70,71,75]. The selective inhibition of USP14 was obtained by using the small-molecule inhibitor IU1–47[59], a more potent derivative of IU1[122], the first selective inhibitor developed for USP14[55]. The new compound is well tolerated in iNeurons (Supplementary Figure 1 A) or SH-SY5Y cells (Supplementary Figure 1A), and in hippocampal and cortical murine primary neurons[59,123].

We used an unbiased TMT-based proteomics approach to evaluate the effect of USP14 inhibition by IU1–47 or KO on the total proteome of iNeurons with the aim of identifying targets of USP14 that may account for its protective effect. A general decrease of the mitochondrial proteome was observed in samples treated with the inhibitor, affecting all three mitochondrial sub-compartments (OMM, IMM, and Matrix) (Fig. 1B–C). The degradation-promoting effect of USP14 inhibition was specific for mitochondria in that other organelles were not reduced in abundance by the treatment (Fig. 1D). GO analysis confirmed these results, and in line with previous literature, highlighted the potential role of USP14 inhibition in several neurodegenerative pathways (Fig. 1E) [91].

By using a combination of biochemical and imaging approaches, we next showed for the first time in iNeurons the selective autophagic and mitophagic effect of USP14 inhibition. The effect on autophagy enhancement, represented by an increased ratio between the lipidated (LC3II) and unmodified form (LC3I) of LC3, was readable after 24 H treatment with IU1–47, and further increased when cells were co-incubated with bafilomycin, indicating that USP14 inhibition does not lead to a blockage of the autophagic flux, but rather enhances autophagy (Fig. 2A). Moreover, the autophagic effect of IU1–47 was abrogated in USP14 KO background, supporting the high specificity of IU1–47 for USP14 (as also seen in previous studies[59,122] (Fig. 2B). Importantly, the effect of IU1–47 on autophagy enhancement was perpetuated in Parkin and PINK1 KO background (Fig. 2C–D), indicating that the autophagic effect of USP14 inhibition is PINK1/Parkin independent. In a similar fashion, we found that the mitophagic flux was enhanced in IU1–47 treated iNeurons, independently of the canonical PINK1/Parkin pathway (Fig. 3A–D). To dissect the molecular pathway underlying the autophagic/mitophagic effect of USP14 inhibition that appeared to be PINK1/Parkin-independent, we turned our attention to alternative autophagic receptors and/or E3 ubiquitin ligases that have been linked to Parkin-independent mitophagy before, namely BNIP3L/NIX[92], and ubiquitin ligases MUL1[93,95,96] and MARCH5[99,124]. We differentiated iNeurons from BNIP3L/NIX KO, MUL1 KO and MARCH5 KO hESCs, treated them with IU1–47, and first measured autophagy levels by assessing the ratio between LC3II and LC3I. We found that IU1–47 induced autophagy (represented by increased LC3II/LC3I) in BNIP3L/-NIX KO and MUL1 KO neurons, but not in the MARCH5 KO background (Fig. 4A–C). We next measured mitophagy in MARCH5 KO iNeurons treated with IU1–47, and found that mitophagy induced by USP14 inhibition was abolished in MARCH5-deficient cells (Fig. 4D–E).

MARCH5 is a RING finger E3 ubiquitin ligase that resides in the outer mitochondrial membrane, and is capable of synthesizing ubiquitin chains via K48 [125], K63[126,127], and K27[128] linkages. It is a key protein for mitochondrial homeostasis, with its activity spanning different layers of the MQC programs. It mediates ubiquitination, and subsequent degradation, of mSOD1 (associated to amyotrophic lateral sclerosis)[129], and polyQ-extended ataxin-3 causing Machado-Joseph disease[130], and suppresses the proteotoxic stress generated by these misfolded proteins in the mitochondria. At the organelle level, MARCH5 maintains optimal mitochondrial morphology and regulates mitochondrial dynamics by impinging on the steady state levels or subcellular localization of core components of the fission and fusion machinery, and their receptors. In particular, MARCH5 seems to inhibit mitochondrial fission via ubiquitination and degradation of pro-fission protein Drp1 [131], Fis1[132], and Drp1 receptor, Mid49[133]. Whereas according to other studies, MARCH5 is required for mitochondrial fission[134], and inhibits mitochondrial fusion by affecting steady state levels of pro-fusion protein Mfn1[135] and Mfn2[136]. MARCH5-mediated K63 ubiquitination of pro fusion and tethering protein Mfn2 facilitates ER-mitochondria interaction[126], a functional feature that is essential for autophagosome formation.

A role of MARCH5 has also been identified in the process of mitochondrial protein import, in opposition to USP30. MARCH5 provides the ubiquitinated substrates that are deubiquitinated by USP30 on the mitochondrial surface to allow their import into mitochondria through the translocase of the outer membrane (TOM)[137]. Accumulation of unimported mitochondrial precursors at the TOM complex activates a stress response that downregulates protein synthesis and upregulates the proteasome[138–140]. This is an essential event that leads to the degradation of unimported ubiquitinated mitochondrial precursors, and eventually activates the mitophagy machinery to eliminate the entire organelle, when severely damaged[141].

Of particular relevance for this work, previous studies identified MARCH5 as a key regulator of mitophagy, with different outcomes depending on cellular context. During hypoxic conditions, MARCH5 targets mitophagy receptor FUNDC1 for ubiquitin-dependent degradation, promoting its degradation, and overall inhibiting FUNDC1-mediated mitophagy[99]. Conversely, MARCH5 downregulation correlates with decreased ubiquitination and degradation of FUNDC1, thus promoting FUNDC1-mediated mitophagy. Because our findings indicate the absolute requirement of MARCH5 for IU1–47-driven mitophagy, and MARCH5 negatively regulates protein levels of FUNDC1, it is unlikely that FUNDC1 operates as mitophagy receptor in the activation of mitophagy induced by IU1–47. Another study describes a pro-mitophagic effect of MARCH5 in that it facilitates mitochondrial recruitment and activity of Parkin on its mitochondrial targets [124]. Our working model however excludes the involvement of Parkin in the mitophagic effect driven by USP14 inhibition.

In our previous work, we showed that the exposure of inner mitochondrial membrane (IMM)-resident protein Prohibitin 2 (PHB2) is indispensable for the mitophagic effect of USP14 inhibition[68]. The molecular mechanism underlying PHB2 exposure during mitophagy, also described in another study[142], is unclear but it requires the translocation of the proteasome to the mitochondrial surface, followed by rupture of the OMM to expose the IMM-resident protein PHB2[68]. Supporting these studies, a recent work described the initiation of a unique form of mitophagy, which does not require the canonical PINK1/Parkin pathway, and depends on the exposure of ubiquitinated IMM-resident proteins to the cytoplasm[143]. On this basis, it is intriguing to hypothesize the existence of a yet unidentified substrate of MARCH5 on mitochondria that can be produced by the MARCH5 ubiquitin ligase to promote mitophagy, and deubiquitinated by USP14 to inhibit mitophagy. Events of ubiquitination promoted by MARCH5 on mitochondria, could promote a remodelling of mitochondrial shape and ultrastructure that results in the exposure of IMM-resident proteins, PHB2 in particular, to promote mitophagy. The physiological relevance of IMM-driven mitophagy is not known; it could be an additional safety mechanism to contain damaged mitochondria, and the ultimate attempt to spare cells from apoptosis (relevant for post mitotic cells like neurons).

Future work will be focused on the identification of ubiquitin-protein conjugates that are synthesized by MARCH5 on mitochondria, and deubiquitinated by USP14.

Finally, as our ultimate goal was to find a way to enhance PINK1/Parkin alternative mitophagy pathway(s) with the long-term perspective to translate the results in the clinical field, it was important for us to understand if the mitophagy boost has a positive impact on the general fitness of mitochondria in Parkin-deficient neurons. In this context, we found that treatment with IU1–47 (5μM– 24 H) rescued the impaired mitochondrial phenotype of PARK2 KO iNeurons (Fig. 6B–C). This result further supported the Parkin-independent mitophagic effect of USP14 inhibition, and the potential clinical application of this approach to enhance general mitochondrial turnover in conditions where this is impaired (e.g., in Parkin-deficient cells). Of note, USP14 inhibition did not have any effect on the overall mitochondrial mass (Fig. 5A–B), nor on mitochondrial respiration of WT cells (Fig. 6B) (as we previously observed in flies[68]). Thus, the mitophagic effect of USP14 inhibition is likely compensated by productive mitochondrial biogenesis. Supporting this hypothesis, we found increased mRNA levels of mitochondrial biogenesis transcription factors PGC1alpha and TFAM (Fig. 5D), and increased mtDNA copy number (Figure S4A) in iNeurons treated with USP14 inhibitor. Importantly, this effect of USP14 inhibition on mitochondrial biogenesis was abrogated in Parkin KO iNeurons (Figure S4B–C). This result suggests that while the mitophagic effect of USP14 inhibition is Parkin-independent, the effect on mitochondrial biogenesis seems to rely on Parkin expression. The molecular mechanism of mitochondrial biogenesis induction by USP14 inhibition is unknown. However, considering the reliance of Parkin expression for this effect, it may be based on Parkin-dependent ubiquitination and degradation of PARIS [46], a negative regulator of mitochondrial biogenesis.

## 5. Conclusion

Approaches that enhance protein and organelle homeostasis can be protective in models of neurodegeneration. Hence, regulation of DUB activity represents a promising target for therapeutic intervention aimed at enhancing mitophagy and mitochondrial turnover. Among this large family of enzymes, USP14 is a particularly appealing target of inhibition because it enhances the UPS, autophagy and mitophagy. Our work shows that USP14 inhibition enhances mitophagy in iNeurons in a PINK1/Parkin-independent fashion, but requires the E3 ubiquitin ligase MARCH5 to execute mitophagy. USP14 inhibition also positively affects mitochondrial biogenesis via an uncharacterized molecular mechanism that requires the expression of Parkin. Overall, USP14 inhibition with specific and potent inhibitor IU1–47 increases both mitochondrial degradation and biogenesis, rejuvenating the mitochondrial repertoire with no apparent toxic effects on neuronal function.

Due to their peculiar architecture, high energetic demands, and post-mitotic state, neurons are particularly vulnerable to the impairment of mitochondrial homeostasis, and difficult to replace. Thus, the coordinated effects that USP14 inhibition exerts on mitophagy and mitochondrial biogenesis are particularly relevant for high metabolic-demand cells such as neurons[144].

Further studies are warranted to explore the involvement of mitochondrial-associated E3 ubiquitin ligase MARCH5 in MQC driven by USP14 inhibition, and assess the physiological relevance of this pathway *in vivo* in murine models of neurodegeneration.

Anexplored effects of USP14 inhibition on neuronal physiology beyond mitochondrial function would also be a valuable follow up of this work. We recently reported that USP14 inhibition rescues the circadian and sleep defects associated to PINK1[69] KO flies. Circadian rhythms are generated by the cyclic expression of clock-controlled genes, and the central pacemaker is identified by a small subset of neurons that in mammals are located in the in the suprachiasmatic nucleus (SCN) of the anterior hypothalamus[145]. It will be interesting to explore the effects of USP14 inhibition on this specific subset of neurons, and clarify the molecular mechanism behind this rescue effect. This is particularly relevant in PD, where among the premotor symptoms, circadian and sleep impairment are most prominent[146–148].

## Supplementary Material

Figure S4

Figure S5

Table S1

Figure S1

Table S2

Table S3

Figure S2

Figure S3

## Figures and Tables

**Materials Table:** 

REAGENTS	SOURCE	IDENTIFIER

**Antibodies**		
anti-LC3A	Novus Biologicals	Cat#NB100-2331
anti-GAPDH	Sigma Aldrich	Cat#G9545-100ul
anti-Vinculin	Sigma Aldrich	Cat#V9264-25ul
anti-TOM20	Santa Cruz Biotechnology	Cat#sc-11415
Anti-Rabbit IgG (H+L), HRP Conjugate	Fisher Scientific	Cat#NA934V
Anti-Mouse IgG (H+L), HRP Conjugate	Fisher Scientific	Cat#NXA931V
Alexa Fluor 488 Goat anti-mouse	Thermo Fisher Scientific	Cat#A10667
Alexa Fluor 488 Goat anti-rabbit	Thermo Fisher Scientific	Cat#A11034
Alexa Fluor 555 Goat anti-mouse	Thermo Fisher Scientific	Cat#A21147
Alexa Fluor 555 Goat anti-rabbit	Thermo Fisher Scientific	Cat#A21430
**Chemicals**		
Oligomycin A	Sigma Aldrich	Cat#O4876
Antimycin A	Sigma Aldrich	Cat#A8674
CCCP	Sigma Aldrich	Cat#C2759
Rotenone	Sigma Aldrich	Cat#R8875
Bafilomycin A	Sigma Aldrich	Cat#B1793
Doxycycline	Sigma Aldrich	Cat#D9891
Y-27632 Dihydrochloride (ROCK inhibitor)	PeproTech	Cat#1293823
Hygromycin B	Thermo Fisher Scientific	Cat#10687-010
Corning Matrigel Matrix, Growth Factor Reduced	Corning	Cat#354230
MitoTracker RED CMX Ros	Thermo Fisher Scientific	Cat#M7512
IU1	Sigma Aldrich	Cat#I1911
IU1-47	Sigma Aldrich	Cat#SML2240
Cycloheximide from Microbial Source	Sigma Aldrich	Cat#C7698
DMEM/F12	Thermo Fisher Scientific	Cat#31331028
Neurobasal	Thermo Fisher Scientific	Cat#21103049
NEAA	Thermo Fisher Scientific	Cat#11140-035
GlutaMax	Thermo Fisher Scientific	Cat#35050038
N-2 Supplement (100X)	Thermo Fisher Scientific	Cat#17502-048
Neurotrophin-3(NT3) Recombinant human	PeproTech	Cat#450-03
Brain-derived neurotrophic factor (BDNF)	PeproTech	Cat#450-02
B27 Supplement	Thermo Fisher Scientific	Cat#17504044
Accutase	Thermo Fisher Scientific	Cat#A1110501
TeSR^™^-E8^™^	StemCell Technologies	Cat#5990
EDTA	Thermo Fisher Scientific	Cat#AM9260G
DMEM Base	Sigma Aldrich	Cat#D5030
HOT FIREPol SolisGreen qPCR mix	Solis BioDyne	Cat#08-46-00001
FuGene HD	Promega	Cat#E2311
MTT	Thermo Fisher Scientific	Cat#M6494
1x Protease Inhibitor Cocktail	Thermo Fisher Scientific	Cat#78442
1xPhosSTOP Phosphatase Inhibitor Cocktail	Thermo Fisher Scientific	Cat#78428
Luminata Forte Western HRP substrate	Thermo Fisher Scientific	Cat#WBLUF0500
**Commercial assays and kits**		Cat#
SensiFast cDNA synthesis kit	Meridian Life Science	Cat#BIO-65054
Pierce BCA Protein assay kits and reagents	Thermo Fisher Scientific	Cat#23227
ReliaPrep RNA Cell Miniprep System	Promega	Cat#Z6011
Seahorse XFe24 FluxPak mini	Agilent Technologies	Cat#102342-100
TMT10plex Isobaric Label Reagent Set plus TMT11-131C Label Reagent	Thermo Fisher Scientific	Cat#A34808
**Oligonucleotides and Recombinant DNA**		
pCMV-hyPBase - hyperactive piggyBac transposase	Sanger Institute	
pAC150-PBLHL-4xHS-EF1a - mtx-QC(mCherry-GFP)XL	Ordureau et al., 2020 [71]	
Primers for PGC1α	5’- GGCAGAAGGCAATTGAAGAG and 5’ - TCAAAACGGTCCCTCAGTTC	
Primers for TFAM	5’ - CCGAGGTGGTTTTCATCTGT and 5’-GCATCTGGGTTCTGAGCTTT	
Primers for GAPDH	5’-GGCCATCCACAGTCTTCTG and 5’-TCATCAGCAATGCCTCCTG	
Primers for Actin	5’-GATCATTGCTCCTCCTGAGC and 5’-ACATCTGCTGGAAGGTGGAC	
Primers for Actin	5’-GATCATTGCTCCTCCTGAGC and 5’-ACATCTGCTGGAAGGTGGAC	
Primers for mtATP6 (mitochondrial DNA)	5’-CGCCACCCTAGCAATATCA and 5’-TTAAGGCGACAGCGATTTC	
Primers for TH (nuclear DNA)	5’-AGGGTATCTGGGCTCTGG and 5’-GGCTGAAAAGCTCCCGATTAT	

## Appendix A. Supporting information

Supplementary data associated with this article can be found in the online version at doi:10.1016/j.phrs.2024.107484.
