# Supplementary figures and images for: USP14 inhibition enhances Parkin-independent mitophagy in iNeurons
Source: Pharmacol Res. Author manuscript; Available in PMC 2026 Jan 28. (PMC12849831; doi:10.1016/j.phrs.2024.107484)

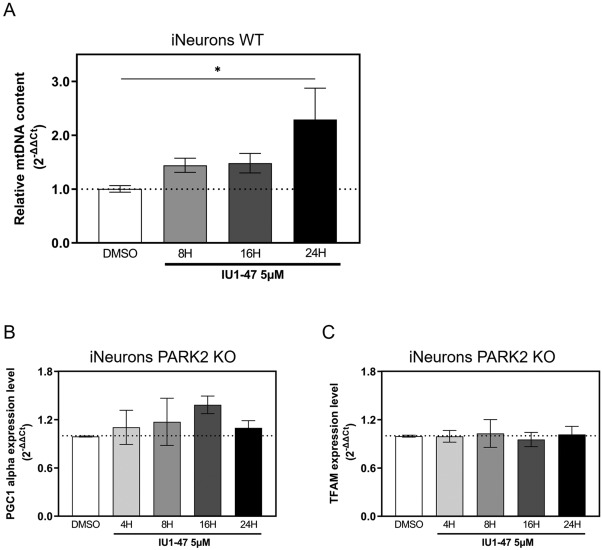

Supplement: Figure S4 [file NIHMS2127424-supplement-Figure_S4.jpg]

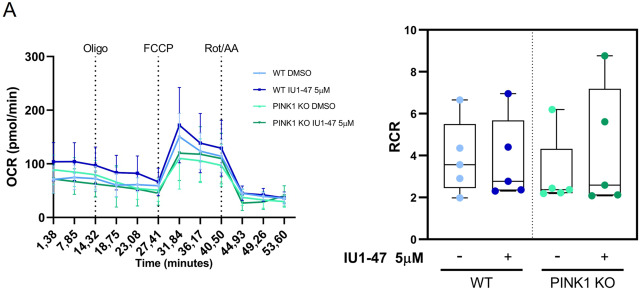

Supplement: Figure S5 [file NIHMS2127424-supplement-Figure_S5.jpg]

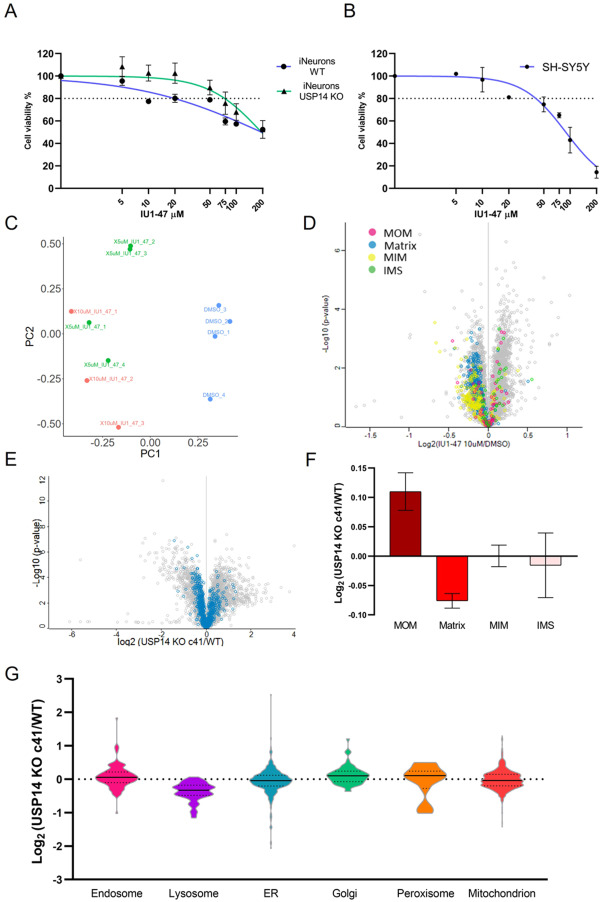

Supplement: Figure S1 [file NIHMS2127424-supplement-Figure_S1.jpg]

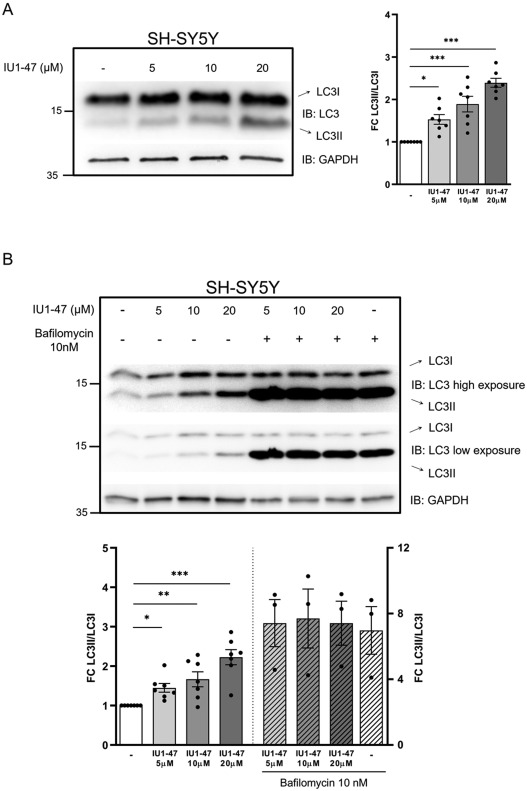

Supplement: Figure S2 [file NIHMS2127424-supplement-Figure_S2.jpg]

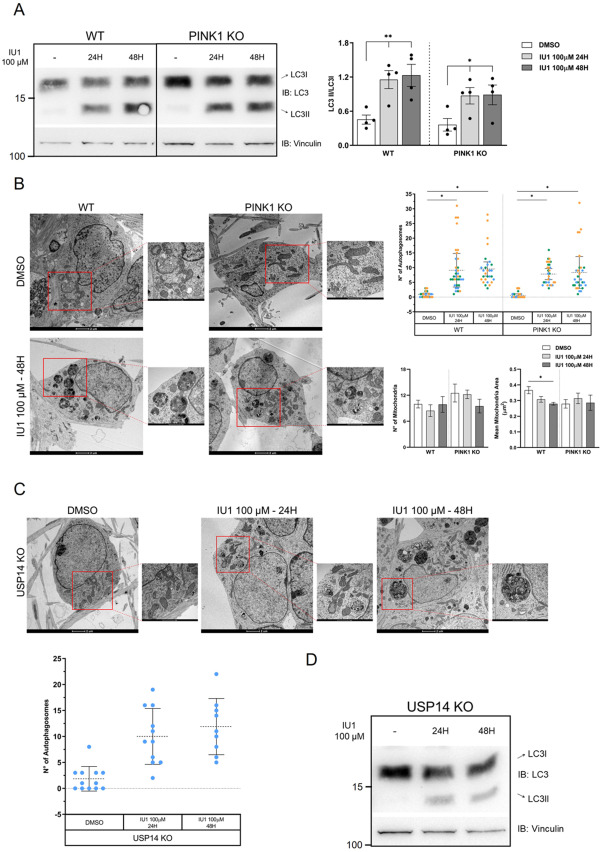

Supplement: Figure S3 [file NIHMS2127424-supplement-Figure_S3.jpg]
